# Driving mechanisms of forest age and climate on carbon sinks in Xinjiang forests

**DOI:** 10.3389/fpls.2026.1775711

**Published:** 2026-03-12

**Authors:** Lili Zhai, Mei Zan, Jia Zhou, Zhongqiong Zhao, Jian Ke, Mao Ye, Ruilian Yuan, Yuting Zhao

**Affiliations:** 1 School of Geographic Science and Tourism, Xinjiang Normal University, Urumqi, China; 2 Xinjiang Laboratory of Lake Environment and Resources in the Arid Zone, Urumqi, China

**Keywords:** climatic factors, forest age, forest NEP, nitrogen neposition, Xinjiang forests

## Abstract

Net ecosystem productivity (NEP) is a key indicator of the carbon sink function of forests, reflecting the combined influence of forest structural characteristics and climatic factors. However, the relative contributions of climate variables to carbon sink formation remain uncertain. This study focuses on forest ecosystems in Xinjiang, China, and develops a statistical regression model relating NEP to forest age over the period 2000-2020. Through residual analysis, the dominant influence of forest age on carbon sinks was effectively isolated. A linear regression model was then applied to quantify the relative contribution of climatic factors to the residual NEP, enabling the decoupling of different driving factors in this region. The results indicate that: (1) Forest age contributes 45-49% to NEP in Xinjiang, confirming its dominant role. (2) The contribution of climatic factors varies with vegetation type, mean annual temperature (MAT) explains a larger proportion of variance (R²= 0.401) in coniferous forests than mean annual precipitation (MAP), whereas broadleaf forests show the opposite pattern, with MAP explaining more variance (R²= 0.399) than MAT. Overall, climatic factors account for 24.52% of the annual total NEP in Xinjiang forests. (3) Decoupling analysis reveals that neglecting forest age may lead to an overestimation of climatic contributions to NEP by approximately tenfold. This approach provides a reliable framework for accurately quantifying the independent effects of different drivers. Considering that younger forests currently constitute a substantial portion of Xinjiang’s forests, they are expected to exhibit a stronger carbon sequestration capacity under current climatic conditions. These findings provide a scientific basis for the precise assessment of forest carbon sink functions in arid regions.

## 1 Introduction

Forests, as one of the most important carbon sinks in terrestrial ecosystems, play an irreplaceable role in regulating the global carbon balance and mitigating climate change due to their strong carbon sequestration capacity (Yu et al., 2024). However, current research on forest carbon sink capacity remains subject to significant uncertainties and complexities. Different models and methodologies often produce markedly divergent estimates of carbon storage and its spatial distribution (Qiu et al., 2020). These discrepancies arise not only from the inherent dynamics and heterogeneity of forest ecosystems but also from the combined influences of natural factors and human disturbances on carbon sink formation, resulting in complex interactions among multiple drivers (Yu et al., 2024; Shan et al., 2025). Therefore, systematically elucidating how these factors interact mechanistically in the carbon sink formation process is critical for overcoming existing limitations in carbon cycle simulation and prediction. A deeper understanding of these intrinsic mechanisms is essential for refining the theoretical framework of the carbon cycle and informing scientifically grounded forest management strategies.

The formation of forest net ecosystem productivity (NEP) is driven by the interplay of multiple factors. Intrinsic biological factors, such as forest type and age structure, directly influence NEP dynamics by regulating vegetation photosynthesis and respiration processes (Leng et al., 2024). Simultaneously, external environmental drivers, particularly climatic factors like temperature and precipitation (Liang et al., 2025; Yi et al., 2010), as well as atmospheric nitrogen deposition (Zhu et al., 2021), exert a significant influence on the spatial distribution of NEP. Existing research indicates that, in mid-to-high latitude regions, mean annual temperature (MAT) is strongly correlated with NEP, whereas in water-limited mid-to-low latitude areas, drought severity emerges as the primary factor explaining NEP variation (Fang et al., 2007; Chen et al., 2025). Notably, many current large-scale NEP estimation models rely heavily on climatic variables, which can underestimate the nonlinear effects of biological factors on carbon flux and introduce uncertainties into regional carbon sink assessments (Carvalhais et al., 2014). For instance, Zhu et al (Zhu et al., 2014). generated a map of China’s maximum potential NEP using only climatic variables such as temperature and precipitation, without accounting for forest biological characteristics. Consequently, these estimates tend to be higher than those derived from inventory data or process-based models (Fang et al., 2007; Wenping et al., 2010; Zhu et al., 2014). This underscores that while climatic factors do influence interannual NEP fluctuations (Dragoni et al., 2011), neglecting the long-term effects of forest age can lead to substantial deviations in carbon sink evaluations (Chen et al., 2003; Xu et al., 2017).

In addition to forest age and climate, atmospheric nitrogen deposition is recognized as a key factor influencing forest carbon sequestration potential. As a critical limiting nutrient for plant growth and photosynthesis, nitrogen can theoretically enhance ecosystem carbon input by promoting vegetation productivity. However, its role in the carbon cycle remains complex and not fully understood. On one hand, the positive effects of nitrogen deposition on ecosystem carbon sinks may be overestimated. While external nitrogen can increase carbon input in the early stages of forest growth, it may also promote the leaching of soluble organic carbon, offsetting or even reversing the carbon sink effect. Furthermore, when nitrogen input exceeds the ecological threshold, nitrogen saturation can occur, leading to nitrate losses via runoff or substantial emissions of N_2_O, thereby weakening carbon retention capacity (Bragazza et al., 2006; Sutton et al., 2008; Schulte-Uebbing et al., 2022; Su et al., 2024). On the other hand, some studies suggest that nitrogen deposition can stimulate trees to allocate more carbon to xylem, producing a positive effect on forest carbon sinks. For example, Reay et al. propose that increasing nitrogen deposition in the future may expand both terrestrial and oceanic carbon sinks. Although the effect on oceanic sinks may be limited, higher nitrogen deposition rates could enhance carbon absorption in northern and tropical forests (Reay et al., 2008). Lin et al. synthesized 408 datasets from global forest ^15^N tracer experiments, highlighting the crucial role of climatic conditions and nutrient supply-demand balance in regulating forest nitrogen retention (Lin et al., 2024). Nevertheless, these conclusions are often derived from correlations between productivity indicators and nitrogen deposition, without fully disentangling the collinear effects of human activity intensity and other nutrient interactions. Additionally, variations in nitrogen deposition responses among different forest ages and tree species may further complicate accurate assessments of its effects on carbon sequestration (Du, 2015).

It is noteworthy that both interannual fluctuations in climatic factors and nutrient inputs from nitrogen deposition ultimately influence NEP through the physiological processes of the forest itself, and their effects are strongly mediated by the forest’s developmental stage. From a plant physiology perspective, forest age is the core endogenous factor determining carbon sequestration rates and the size of forest carbon pools (Du, 2015; Lu et al., 2023; Shan et al., 2025). During the juvenile stage, a high photosynthetically active radiation utilization rate drives rapid canopy expansion, resulting in a large leaf area index and exponential biomass accumulation. As forests mature, respiratory demands increase, and more carbon is allocated to structural tissues such as xylem, producing a unimodal or monotonically decreasing trend in NEP along the forest age gradient (Genet et al., 2009; Li and Liu, 2014). Forest ecosystem productivity therefore exhibits pronounced age-dependent characteristics, a pattern consistently observed across multiple studies (Piao et al., 2009; Coursolle et al., 2012; Jianwu et al., 2014; Gao et al., 2016; Chen et al., 2020). Research indicates that during forest community succession, the influence of initial forest age on the spatial variation of NEP significantly exceeds the effects of climate and long-term environmental changes (Pregitzer and Euskirchen, 2004; Besnard et al., 2018). For example, in Asia, scholars such as Yu (Yu et al., 2014) and Gao (Gao et al., 2016) demonstrated quantitatively that forest age accounts for approximately 42% of the observed variation in NEP within study areas. In recent years, the integration of multi-source data, including site observations, remote sensing, GIS datasets, and process-based model outputs, has further clarified the long-term relationship between NEP and forest age (Zhou et al., 2015; Gao et al., 2016). These findings underscore that neglecting forest age in carbon sink assessments can introduce substantial biases.

However, existing research often does not adequately account for the long-term effects of forest age when examining the relationships between external drivers, such as climate or nitrogen deposition, and NEP. This oversight can lead to confounding effects of forest age structure on the apparent influence of environmental factors. For instance, previous studies have applied age-class grouping to analyze how carbon fluxes respond to external drivers across different forest age groups (Coursolle et al., 2012). Yet, there remains a lack of research that explicitly decouples the effects of environmental factors while controlling for forest age as a baseline. Drawing inspiration from dendroclimatology, which emphasizes that “removing the growth trend effect of trees is essential for accurately assessing the true impact of environmental factors on tree-ring width,” this study focuses on the forests of Xinjiang. Using data from forest survey plots, we apply residual analysis to remove the dominant influence of forest age on carbon sinks, quantitatively assess the relative contributions of climatic factors to forest productivity, and compare the spatial distribution of NEP as determined by climate factors both with and without controlling for forest age.

## 2 Materials and methods

### 2.1 study area

**Figure 1:**
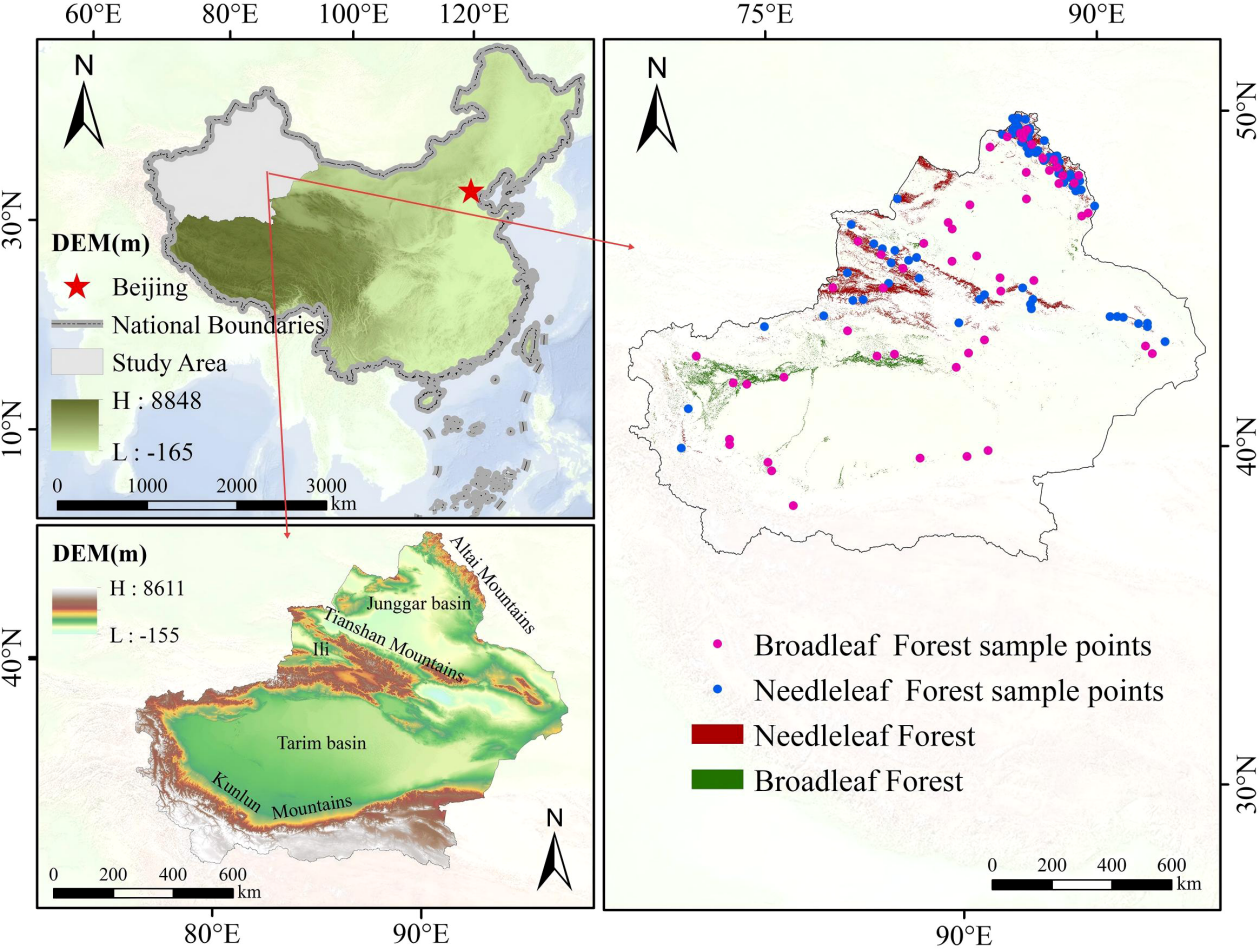
Overview of the study area.

The Xinjiang Uygur Autonomous Region, located on China’s northwest border (73°32′-96°21′E; 34°22′-49°33′N), spans approximately 1,500 km from north to south and 1,900 km from east to west. Covering a total area of 1.66 × 10^4^ km^2^, it accounts for roughly one-sixth of China’s landmass. Situated in the Eurasian hinterland, Xinjiang is characterized by vast terrain, scarce and unevenly distributed precipitation, and a typical inland arid climate. The region’s topography follows a “three mountains and two basins” pattern and is influenced by both westerly circulation and the edge effects of plateau monsoons, resulting in a temperate continental arid-semi-arid climate (Chen et al., 2023). Xinjiang’s forested area totals approximately 8.3 × 10^4^ km², primarily located in river valleys such as the Altai Mountains, Tianshan Mountains, Ili River, Ertix River, and Tarim River, as well as along oasis margins. The main forest types include evergreen needleleaf forest (ENF), deciduous needleleaf forest (DNF), deciduous broadleaf forest (DBF), and the less common mixed forest (MF). Because DNF and MF are relatively scarce, this study groups forests into two categories, coniferous and broadleaf, when comparing modeling differences before and after removing the forest age trend, with MF merged into the broadleaf category (**Figure 1**). Coniferous forests are dominated by Picea schrenkiana and Siberian larch, primarily distributed across the Tianshan and Altai Mountains. Broadleaf forests are dominated by Populus euphratica, mainly found in the Tarim River basin, characterized by rapid growth and a relatively young mean tree age (Hu et al., 2005).

### 2.2 Data acquisition and processing

#### 2.2.1 Meteorological data, atmospheric nitrogen deposition and NEP data

In this study, meteorological data primarily include temperature and precipitation, both obtained from the National Earth System Science Data Center (http://loess.geodata.cn) (Peng et al., 2019). These datasets are derived from CRU and WorldClim climate data and were downscaled to the China region using the Delta spatial downscaling method, achieving a spatial resolution of 1 km. MAT and mean annual precipitation (MAP) for Xinjiang during 2000–2020 were calculated by averaging the respective temperature and precipitation data over this period.

This study integrates two datasets to construct an atmospheric nitrogen deposition dataset for Xinjiang during 2000-2020. The first dataset, compiled by Jia et al (Jia et al., 2021), is based on site-year observations of atmospheric inorganic deposition (http://www.nesdc.org.cn) obtained through literature review. Using Kriging spatial interpolation, a continuous nitrogen deposition dataset for China from 1996 to 2015 was generated, with a spatial resolution of 1 km. The second dataset, provided by Professor Zhao Yu’s team at Nanjing University, covers nitrogen deposition from 2005 to 2020. It integrates multiple sources, including ground observations, chemical transport model simulations, satellite vertical column densities, and meteorological and geographic variables, and uses random forest (RF) and generalized additive models for estimation. The spatial resolution of this dataset is 0.25° × 0.25° (Zhou et al., 2023). Due to the differences in spatial resolution between the two datasets, the dataset by Zhao Yu et al. was first downscaled to 1 km to match the spatial baseline of Jia et al.’s dataset. To eliminate systematic bias between the datasets, a correction coefficient (CC) was calculated using the overlapping period (2005-2015) as follows: *CC* = *H*_2005-2015/_*F*_2005-2015_, where *H*_2005–2015_ is the spatial arithmetic mean of nitrogen deposition from Jia et al. during 2005-2015, and *F*_2005–2015_ is the corresponding mean from Zhao Yu et al. This CC was then applied to the dataset by Zhao Yu et al. (2005-2020) to remove systematic bias: *F_t_′* = *F_t_*× CC, where *F_t_′* represents the nitrogen deposition raster for year *t* after bias correction, *F_t_*​ is the original raster data for year *t*; and *t* ranges from 2005 to 2020.

NEP represents the portion of total primary productivity (GPP) remaining after accounting for autotrophic and heterotrophic respiration as well as the consumption of photosynthetic products. NEP products were derived using MCD12Q1 data based on the international geosphere-biosphere program classification standard, combined with MCD43A4 data to obtain normalized difference vegetation index (NDVI) and enhanced vegetation index products. RF algorithms were applied to estimate NEP (http://www.geodata.cn). Validation against surface flux tower measurements (FluxNET) indicates good accuracy, with R² = 0.74 and RMSE = 128.35 g C m^-^² yr^-^¹. The data have an annual temporal resolution and a spatial resolution of 1 km. To ensure consistency across all datasets, the NEP data were resampled to a 500 m resolution.

#### 2.2.2 Forest age and forest distribution data

The forest age data used in this study were derived from the 2000–2020 Xinjiang forest age dataset developed by our research team. This dataset combines forest survey data and remote sensing observations. First, a RF model was used to estimate forest age in Xinjiang for 1991. Next, using Landsat time-series data from 1991-2022, the LandTrendr detection algorithm on the google earth engine platform was applied to identify forest loss and gain events between 1992 and 2022. By integrating these forest disturbance and regrowth events, a spatially explicit map of forest age in Xinjiang from 1991 to 2022 was generated. Validation of the RF model indicated an R² greater than 0.65, with relative root mean square error (RRMSE) ranging from 0.119 to 0.183. The overall accuracy for detecting forest disturbance and gain exceeded 85%, demonstrating the high reliability of this dataset. These data were subsequently used to estimate NEP in the study area (Zhai et al., 2025). Forest type and distribution information were obtained from the National Forestry Department’s forest inventory dataset.

#### 2.2.3 Forest plot data

The forest plot data for this study were primarily obtained from the Xinjiang Forest Resource Investigation, which includes a Type I inventory and a Type II survey. The Type I inventory employs systematic sampling and remote sensing to provide a broad assessment of forest resources, documenting land types, forest ages, areas, and dominant tree species (Zan, 2016). The Type II survey provides detailed information at the plot level, including forest age, diameter at breast height, and dominant tree species. In this study, forest age data from selected Type II plots were primarily used to construct models relating forest age to NEP.

For each forest plot, the annual average values of NEP, MAP, MAT, and nitrogen deposition were extracted from a 3 × 3 pixel window centered on the plot coordinates and then averaged. These averaged values were assigned as the corresponding factor values for that plot.

### 2.3 Research methods

#### 2.3.1 Residual analysis method

Residuals represent the differences between observed values and the values predicted by a model, reflecting the variation not explained by the model. Essentially, they correspond to the estimated error terms, expressed as: $e_{i}=y_{i}-{\hat{y}}_{i}$, where $e_{i}$ is the residual for the *i* -th observation, $y_{i}$ is the observed value of the dependent variable for the *i*-th observation, ${\hat{y}}_{i}$ and is the predicted value for the *i* -th observation (Zuur et al., 2010). In this study, the NEP residual is defined as the difference between the actual NEP and the NEP simulated by the corresponding model.

This study aims to establish a ‘forest age-climate’ decoupling analysis framework. The influence of forest age is first removed from the NEP data extracted from corresponding plots, after which the contribution of climate factors to NEP is quantitatively assessed. Since NEP is determined by the combined effects of forest structure (forest age) and abiotic factors (climate), residual analysis is employed to independently quantify the relative contributions of these drivers. Specifically, a statistical model is first constructed to evaluate the contribution of forest age to NEP over long timescales, and the NEP residual, representing the portion not explained by forest age, is calculated. Subsequently, the relative contribution of climate factors is assessed using a regression model linking the NEP residuals to climatic variables.

#### 2.3.2 Statistical analysis methods

All statistical analyses in this study were conducted using SPSS 27. Pearson correlation analysis was performed to evaluate the bivariate relationships between potential predictor variables, forest age, MAP, MAT, and nitrogen deposition, and NEP for coniferous and broadleaf forest plots (**Tables 1**, **2**). The ‘Mark Significant Correlation Coefficients’ option was used to automatically annotate significance levels next to the correlation coefficients (* for p < 0.05, ** for p < 0.01), allowing quantification of both the strength and statistical significance of the linear relationships between variables.

**Table 1:** Pearson correlation coefficient matrix between coniferous forest variables.

	Age	MAP	MAT	Ndep	NEP
Age	1	0.145	-0.511	0.490**	-0.644**
MAP		1	-0.104	-0.157	-0.238
MAT			1	0.438*	0.612**
Ndep				1	0.641*
NEP					1

* for p<0.05; ** for p<0.01;.

Ndep is the abbreviation of Nitrogen deposition.

**Table 2:** Pearson correlation coefficient matrix between broadleaf forest variables.

	Age	MAP	MAT	Ndep	NEP
Age	1	-0.62*	-0.501**	-0.374**	-0.616**
MAP		1	-0.126	0.419*	0.624**
MAT			1	0.284	0.377*
Ndep				1	0.637*
NEP					1

(Related comments are the same as **Table 1**).

A path analysis approach based on multiple regression was employed to construct a series of equations quantifying the direct and indirect effects among variables. In this framework, the total effect of an independent variable on a dependent variable is decomposed into the direct effect, which represents the immediate influence, and the indirect effect, which operates through mediator variables. This method effectively evaluates the relative importance of different pathways and is well-suited for analyzing moderately complex causal models containing three to five variables. It provides clear insights into the potential mechanisms linking the variables (Adzrago and Williams, 2023). The direct path coefficient (CoeffDP) represents the standardized partial regression coefficient of the predictor variable on NEP, while the indirect path coefficient (CoeffIP) is calculated as ‘correlation coefficient (Pearson) × CoeffDP,’ reflecting the transmission effect between variables.

#### 2.3.3 NEP estimation method using MAT and MAP

To compare NEP estimation results, this study applied the optimal estimation method proposed by Zhu et al (Zhu et al., 2014), which estimates potential vegetation carbon flux in China using MAT and MAP from meteorological stations, along with their interaction, without accounting for biological factors. The approach is defined as follows: GEP=107.02×MAT+2.18×MAP−0.1×MAT×MAP−544.35, ER=0.68×GEP+81.90, NEP=GEP−ER, where GPP represents the gross primary production of the ecosystem, and ER denotes ecosystem respiration. Using this method, combined with meteorological data from Xinjiang and forest plot data, the contribution of climate factors to carbon flux in Xinjiang’s forests was estimated without removing the forest age effect. These results were then compared with NEP simulated by the model after removing the forest age trend, allowing the differences in climate-driven NEP before and after accounting for forest age to be evaluated.

### 2.4 Research framework and process

**Figure 2:**
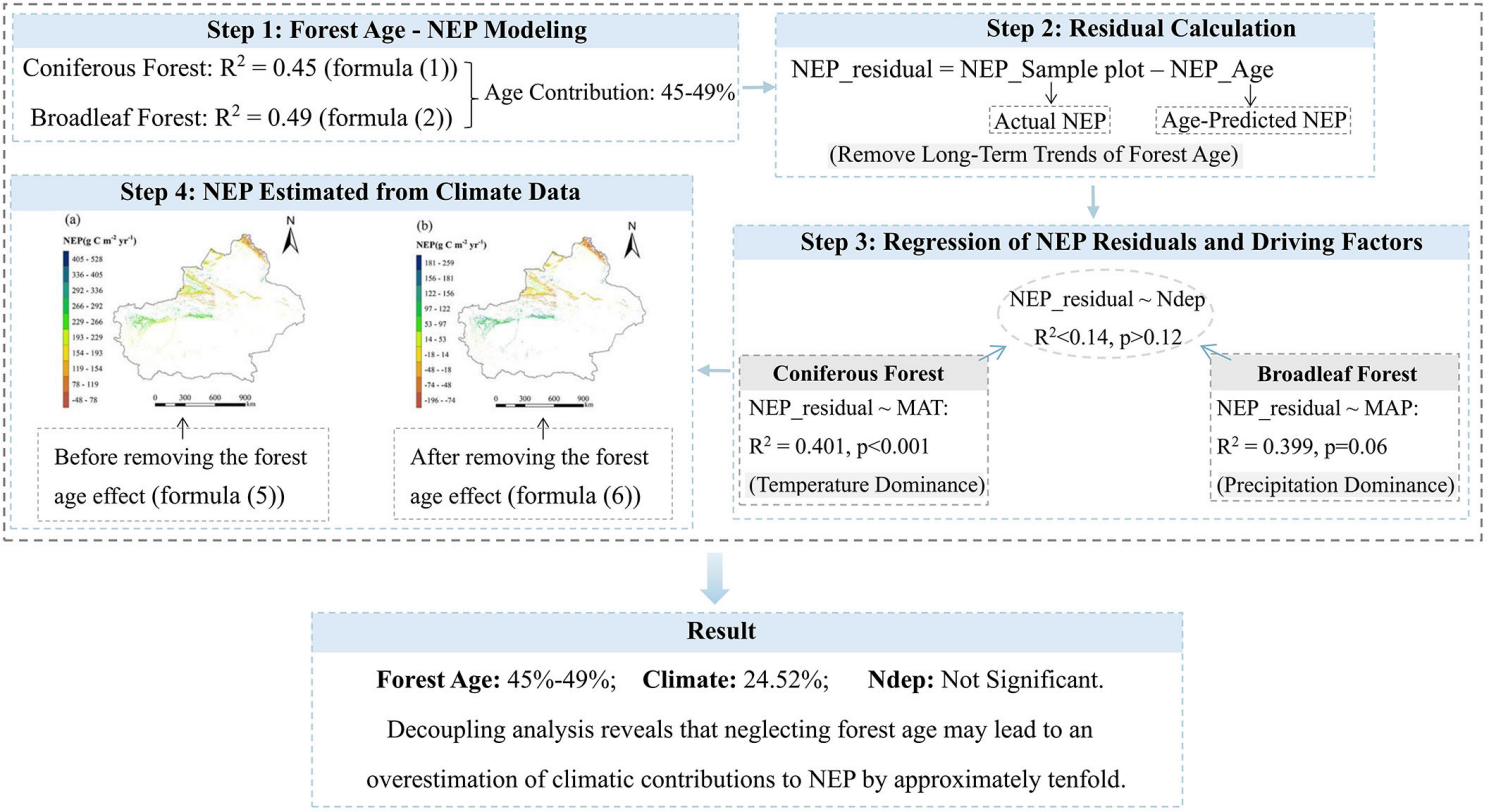
Flowchart for this study.

This study employs the following three steps to assess the contributions of forest age, climate, and nitrogen deposition to the net ecosystem productivity (NEP) of forests in Xinjiang (**Figure 2**): (1) A statistical regression model was constructed to analyze the relationship between forest NEP and forest age for coniferous and broadleaf forests in Xinjiang from 2000 to 2020, allowing for the determination of the contribution rate of forest age to NEP; (2) The NEP residuals for the forest plots were obtained by calculating the difference between the actual NEP and the NEP simulated based on forest age, thereby removing the long-term trend of forest age effects; (3) linear regression analysis on the calculated NEP residuals with temperature, precipitation, and nitrogen deposition for coniferous and broadleaf forests; (4) Calculate the spatial distribution of Xinjiang forest NEP estimated by climatic factors before and after removing the influence of forest age. Finally, quantify the relative contributions of each influencing factor to forest NEP, thereby decoupling the contributions of different driving factors in the region.

## 3 Analysis of results

### 3.1 Correlation between potential predictors and NEP

Pearson correlation analysis indicates that, in Xinjiang’s coniferous forests, both MAT and forest age have significant effects on NEP (p < 0.01), while nitrogen deposition is moderately correlated with NEP (p < 0.05). In contrast, MAP does not show a significant correlation with NEP (**Table 1**). For broadleaf forests, MAP and forest age are significantly correlated with NEP (p < 0.01), and nitrogen deposition is moderately correlated (p < 0.05), whereas MAT does not exhibit a significant relationship with NEP (**Table 2**).

Path analysis of the stepwise regression model for Xinjiang’s coniferous forests shows that the final model includes only MAT and forest age. This indicates that both variables not only exert direct effects on forest NEP but also influence other factors, generating indirect pathways affecting NEP (**Table 3**). Similarly, for broadleaf forests, path analysis identifies only MAP and forest age in the final regression equation, suggesting that these variables may indirectly regulate NEP dynamics through interactions with other ecological factors in broadleaf forest ecosystems (**Table 4**).

**Table 3:** Coniferous forest path analysis results.

				CoeffIP	
PV	C_NEP-_*_x_*	C_MAT-Age_	CoeffDP	MAT	Age
MAT	0.612	-0.318	0.509	/	0.219
Age	-0.644	-0.318	-0.429	0.26	/

C_NEP-_*_x_*is the degree of correlation between NEP and influencing factors.

C_MAT-Age_ is the degree of correlation between age and MAT.

The threshold value of the variable input model is set to the probability value of the F test equals 0.1 (p ≤ 0.1).

PV, CoeffDP and the abbreviations of CoeffIP are Predictor variable, Coefficient of direct path and coefficient of indirect path respectively.

**Table 4:** Broadleaf forest path analysis results.

				CoeffIP	
PV	C_NEP-_*_x_*	C_MAP-Age_	CoeffDP	MAP	Age
MAP	0.624	-0.62	0.607	/	0.295
Age	-0.616	-0.62	-0.477	0.376	/

C_MAP-Age_ is the correlation coefficient between MAP and age.

(Other abbreviations are the same as **Table 3**).

### 3.2 The relationship between forest age and NEP

**Figure 3:**
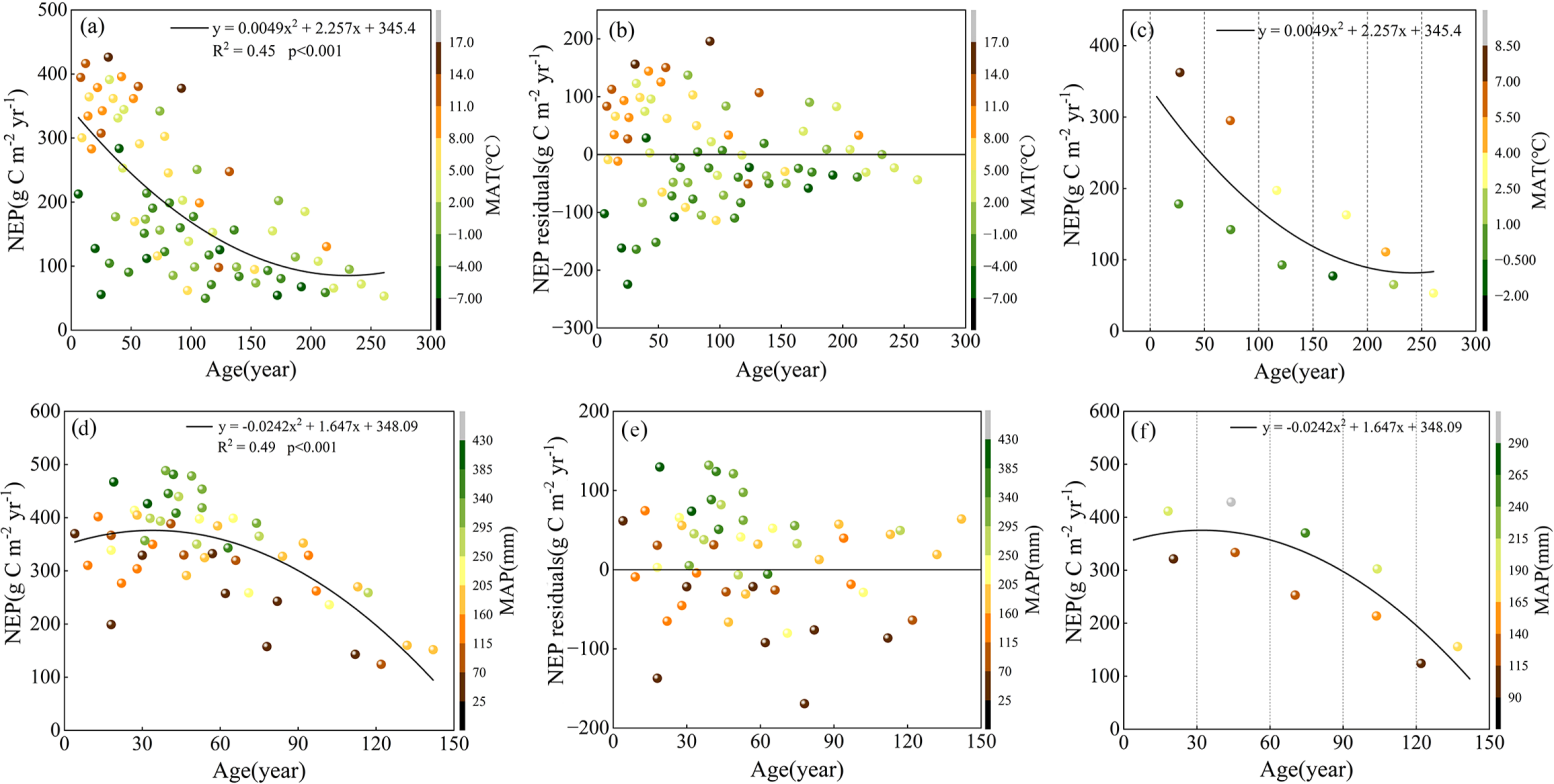
**(a)** The correlation between NEP of coniferous forest and forest age (n = 76); **(b)** The correlation between NEP residuals (observed values minus the corresponding simulated NEP) and forest age for coniferous forest; **(c)** Highlighting the impact of MAT on NEP for different age groups. The color of the points in **(a–c)** represents the temperature of the coniferous forest plots. **(d)** The correlation between NEP of broadleaf forest and forest age (n = 53); **(e)** The correlation between NEP residuals (observed values minus the corresponding simulated NEP) and forest age for broadleaf forest; **(f)** Highlighting the impact of MAP on NEP for different age groups. The color of the points in **(d–f)** represents the precipitation of the broadleaf forest plots.

The statistical analysis of NEP and forest age for Xinjiang’s coniferous and broadleaf forests indicates that NEP is significantly correlated with forest age in both forest types. In coniferous forests, forest age independently explains 45% of the variation in NEP (**Figure 3a**), and the corresponding regression model is represented by Equation 1. In broadleaf forests, forest age accounts for 49% of the NEP variation (**Figure 3d**), with the associated regression equation given by Equation 2. **Figures 3b, c, e, f** further illustrate the relationship between NEP residuals and stand age as well as climate factors, which is explained in detail in Section 3.3.

(1) $$NEP=0.0049\times a{\text{ge}}^{2}-2.257\times a\text{ge}+345.4(R^{2}=0.45,p<0.001)$$

(2) $$NEP=-0.0242\times a{\text{ge}}^{2}+1.647\times a\text{ge}+348.09(R^{2}=0.49,p<0.001)$$

### 3.3 Compare the relationship between climate factors and NEP before and after considering the forest age trend

Based on the statistical analyses presented in Section 3.1, statistical regression models of NEP driven by climatic factors were constructed separately for coniferous and broadleaf forests in Xinjiang. For coniferous forests, a regression model between MAT and NEP was established (Formula (3)), showing that MAT explains 49.60% of the variation in NEP. For broadleaf forests, a regression model between MAP and NEP was developed (Formula (4)), with MAP accounting for 52.20% of the variation in NEP.

(3) $$NEP=12.57\times MAT+154.45(R^{2}=0.496,p<0.001)$$

(4) $$NEP=0.63\times MAP+197.6(R^{2}=0.522,p<0.001)$$

Because forest carbon sequestration is closely linked to forest age, this study removed the forest age trend from NEP data to more accurately assess the contribution of climate and to minimize interference from the dominant influence of stand age over long timescales. Using Equations 1 and 2, the NEP simulated by forest age was calculated for all coniferous and broadleaf forest plots in Xinjiang. The difference between the observed NEP and the simulated NEP was defined as the NEP residual, representing the portion of NEP not explained by forest age. As shown in **Figures 3b and e**, the NEP residuals are no longer associated with stand age. For coniferous forests (**Figure 3b**), residuals generally increase with rising temperatures, indicating that sites with higher temperatures tend to have higher NEP residuals. For broadleaf forests (**Figure 3e**), NEP residuals increase with increasing precipitation. These patterns suggest that, after accounting for forest age, temperature and precipitation exert additional influence on the NEP of coniferous and broadleaf forests, respectively. To further validate these findings, plots were grouped by age: coniferous forests into six age classes (0-50, 50-100, 100-150, 150-200, 200-250, and 250–300 years) and broadleaf forests into five age classes (0-30, 30-60, 60-90, 90-120, and 120–150 years). For each group, average stand age, NEP, MAT (**Figure 3c**), and MAP (**Figure 3f**) were calculated for plots above and below the fitted curves. The results demonstrate that, at similar stand ages, MAT and MAP remain significant drivers of NEP in coniferous and broadleaf forests, respectively.

**Figure 4:**
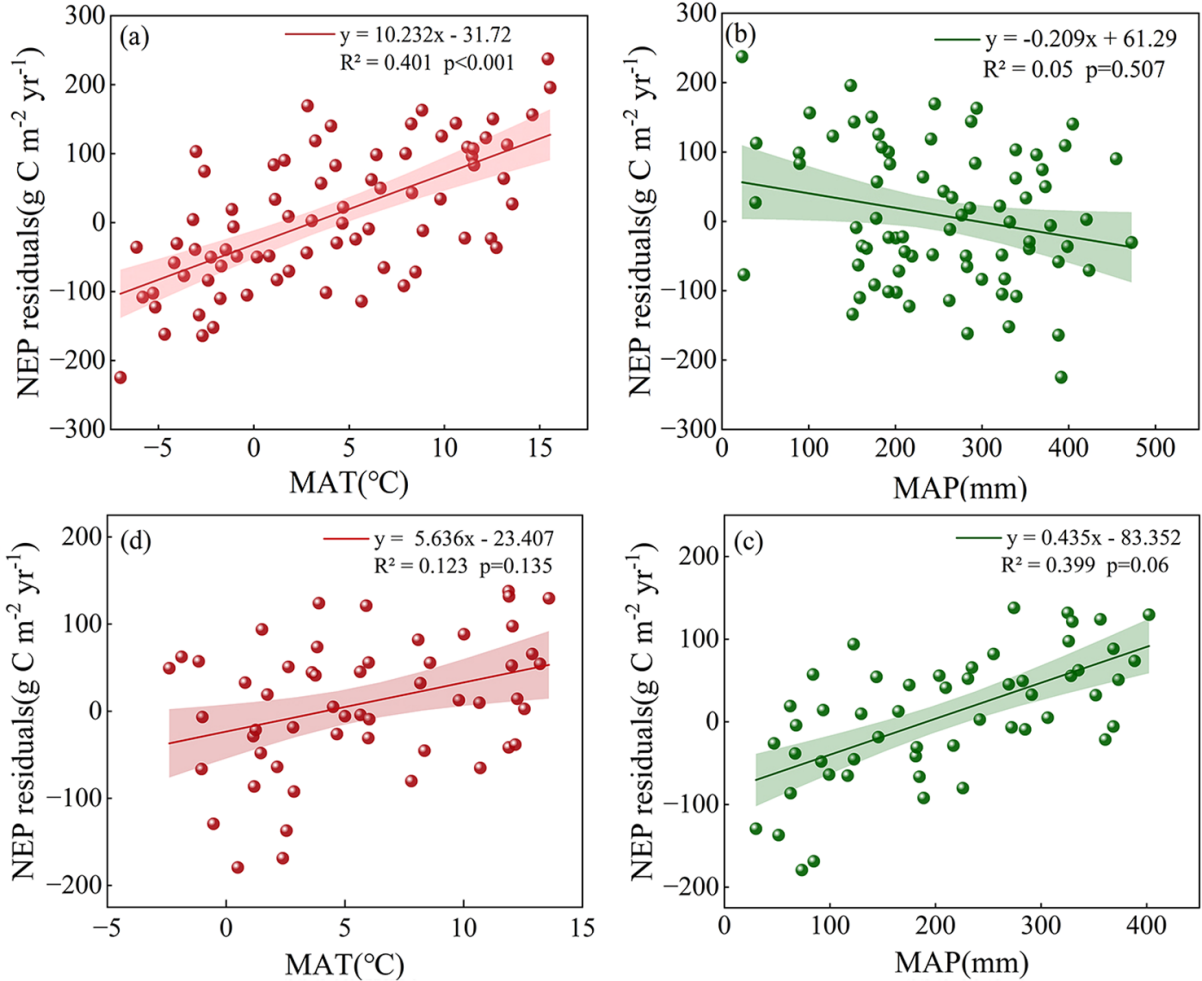
**(a)** Relationship between NEP residuals in coniferous forest and MAT; **(b)** Relationship between NEP residuals in coniferous forest and MAP. **(c)** Relationship between NEP residuals and MAT in broadleaf forests; **(d)** Relationship between NEP residuals and MAP in broadleaf forests.

After removing the influence of stand age on NEP, regression analyses were conducted between the NEP residuals of coniferous and broadleaf forests and MAT and MAP, respectively. The results show that NEP residuals of coniferous forests are positively correlated with MAT, while NEP residuals of broadleaf forests are positively correlated with MAP (**Figures 4a, c**). These relationships are represented by Equations 5 and 6. The models indicate that MAT explains 40.1% of the variation in coniferous forest NEP residuals, whereas MAP accounts for 39.9% of the variation in broadleaf forest NEP residuals. Additional analyses were conducted to examine the relationships between NEP residuals of coniferous forests and MAP, as well as NEP residuals of broadleaf forests and MAT. These analyses revealed no significant relationships, with R² = 0.05 for coniferous forests versus MAP and R² = 0.123 for broadleaf forests versus MAT (**Figures 4b, d**). These results highlight the strong vegetation-type dependence of the climatic controls on forest NEP after accounting for forest age.

(5) $$NEPresiduals=10.131\times MAT-31.72(R^{2}=0.401,p<0.001)$$

(6) $$NEPresiduals=0.435\times MAP+83.352(R^{2}=0.399,p=0.06)$$

### 3.4 Effects of nitrogen deposition on NEP

**Figure 5:**
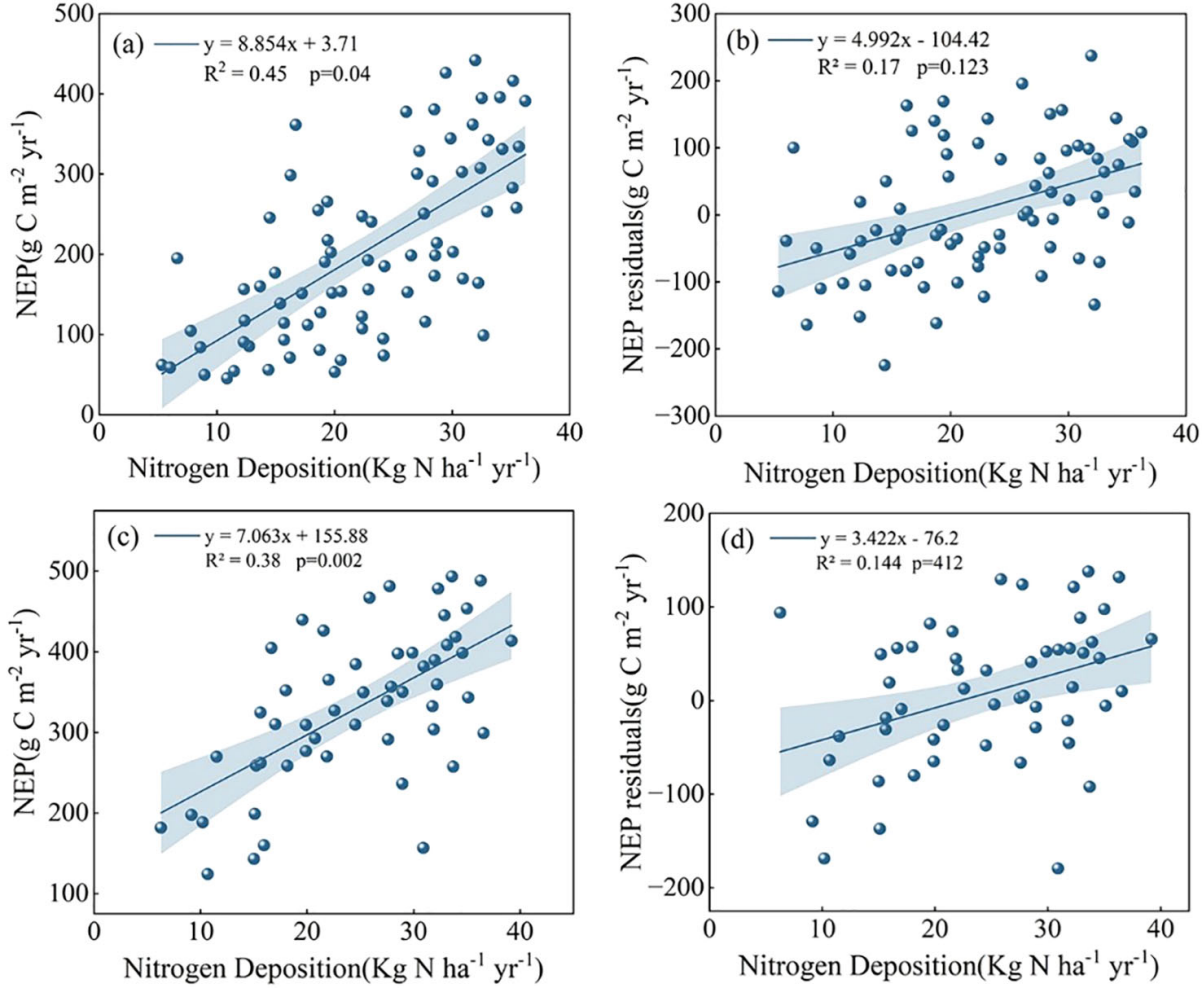
**(a)** Relationship between NEP and nitrogen deposition in coniferous forests; **(b)** Relationship between NEP residuals and nitrogen deposition in coniferous forests. **(c)** Relationship between NEP and nitrogen deposition in broadleaf forests; **(d)** Relationship between NEP residual and nitrogen deposition in broadleaf forests.

As a potential driver of forest NEP, the mechanisms by which nitrogen deposition affects NEP require further clarification. In this study, when the forest age effect was not removed, NEP in both coniferous and broadleaf forests was significantly correlated with nitrogen deposition (p < 0.05), with explained variances of 0.45 and 0.38, respectively (**Figures 5a, c**), consistent with previous studies. However, after removing the forest age trend, the correlation between NEP residuals and nitrogen deposition weakened substantially (R² < 0.2) and was no longer statistically significant (**Figures 5b, d**). It is important to note that this result does not imply that nitrogen deposition has no effect on forest carbon sinks; rather, it suggests that, at the regional scale, nitrogen deposition may not be the most reliable predictor of spatial variation in NEP.

### 3.5 Xinjiang forest NEP estimated from climate data

**Figure 6:**
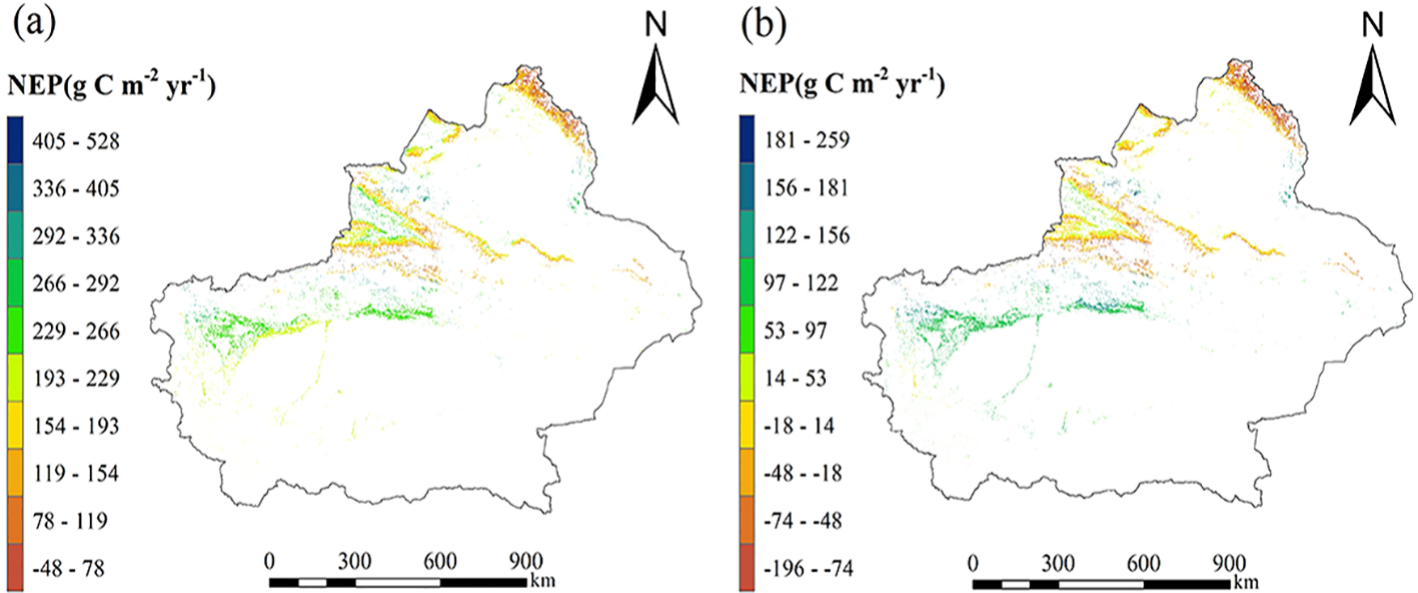
**(a)** The spatial distribution of forest NEP in Xinjiang estimated using climate factors (MAT and MAP) before removing the forest age effect. **(b)** Xinjiang forest NEP spatial distribution derived from climatic variables after removing the forest age effect.

Using meteorological data from Xinjiang for 2000-2020, forest distribution data, and the statistical relationships between coniferous forest NEP and its residuals with MAT, as well as broadleaf forest NEP and its residuals with MAP, the spatial distribution of forest NEP in Xinjiang was estimated (**Figure 6**). Without removing the forest age effect, NEP estimated solely from climate factors (Equations 3, 4) is shown in **Figure 6a**, with an average NEP of 190.734 g C m^-2^ yr^-1^, and a total annual NEP of 159.520 Tg C. After removing the dominant influence of forest age, the average NEP residual estimated using the regression model between NEP residuals and climate (Equations 5, 6) was 20.569 g C m^-2^ yr^-1^, with a total annual NEP residual of 17.140 Tg C (**Figure 6b**). These results indicate that neglecting the effect of forest age and relying solely on climate factors substantially overestimates the contribution of climate to carbon sinks. When combined with the age-related variation of forest carbon sinks, it is evident that NEP in the region is significantly influenced by climate. Spatially, NEP residuals show a general south-to-north decline, with positive NEP concentrated in the Tarim River Basin and Ili River Valley, whereas negative NEP is mainly observed in the Altai Mountains and eastern Tianshan Mountains.

**Figure 7:**
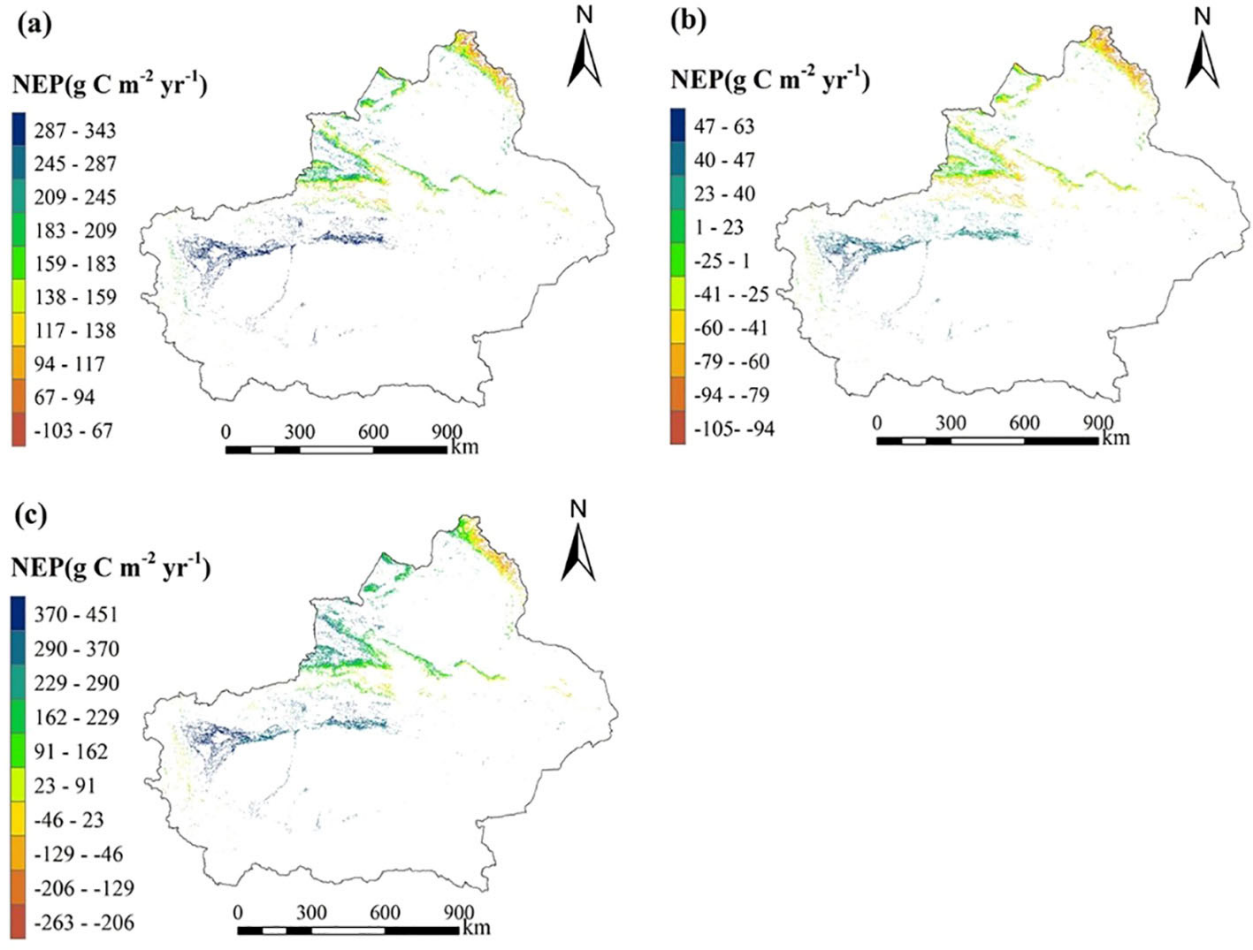
**(a)** The spatial distribution of NEP before removing the forest age effect trend without distinguishing forest types (estimated using Equation 7); **(b)** The spatial distribution of NEP residuals after removing the forest age effect trend without distinguishing forest types (estimated using Equation 7); **(c)** The spatial distribution of NEP estimated only using climate (estimated using Equation 10).

To further compare estimation outcomes, all coniferous and broadleaf forest plots were analyzed together, without distinguishing forest types, to examine the relationship between NEP and climatic factors. Based on this combined analysis, the spatial distribution of forest NEP in Xinjiang was estimated and compared with results obtained when forest types were distinguished. When forest types were not separated, NEP showed a strong correlation with MAT and forest age (p < 0.05), while the correlation between MAP and NEP was less pronounced. The regression correlation between NEP, forest age, and MAT is represented by Equations 7 and 8, and the relationship between NEP residuals and MAT is described by Equation 9. Using Equation 8, the average NEP of Xinjiang forests was estimated at 202.180 g C m^−2^ yr^−1^, with a total annual NEP of 168.500 Tg C (**Figure 7a**). The average NEP residual estimated by Equation 9 was -9.720 g C m^−2^ yr^−1^, with a total annual NEP residual of -8.100 Tg C (**Figure 7b**). These results indicate that, without distinguishing forest types, the apparent contribution of climate factors to carbon sinks is substantially increased when the forest age effect is not removed, yielding total NEP estimates similar to those obtained when forest types are separated. However, the negative values of the NEP residuals and the notable differences from type-specific results underscore the importance of accounting for forest type. Given the significant variations in terrain and hydrothermal conditions across the primary distribution areas of coniferous and broadleaf forests in Xinjiang, analyses that distinguish forest types provide more accurate NEP estimates.

(7) $$NEP=0.0046\times {\text{age}}^{2}-2.334\times \text{age}+354.3(\text{FFFD;}R^{2}=0.4,p<0.001)$$

(8) $$NEP=11.98\times MAT+155.29(R^{2}=0.51,p=0.003)$$

(9) $$NEPresiduals=6.863\times MAT-44.96(R^{2}=0.235,p<0.01)$$

This study also applied the optimal estimation method of Zhu et al (Zhu et al., 2014). (Equation 10) to evaluate the maximum potential of forest NEP in Xinjiang. This method considers only climate factors and does not account for the influence of forest age. From the estimated spatial pattern of forest NEP (**Figure 7c**), the average NEP is 198.728 g C m^-2^ yr^-1^, with a total annual NEP of 165.569 Tg C. These results are similar to the NEP estimates obtained in this study without removing the forest age effect, but they are substantially higher than estimates after accounting for forest age trends. This suggests that Zhu et al.’s method is appropriate for assessing the maximum potential of forest NEP in Xinjiang when the influence of forest age is not removed.

(10) NEP=34.25×MAT+0.7×MAP−0.03×MAT×MAP−92.29

## 4 Discussion

### 4.1 Mechanism of influence of different factors on NEP

This study compared forest NEP estimation in Xinjiang using climate factors before and after removing the effect of forest age. When the forest age effect was not removed, the fit between climate variables and NEP was stronger, largely because forest age is closely associated with NEP. After removing the forest age effect, the R² values for MAT and MAP with coniferous and broadleaf forest NEP both decreased by approximately 0.1. These findings further highlight that forest age is a major driver of NEP in Xinjiang (R² = 0.45 for coniferous forests and R² = 0.49 for broadleaf forests), underscoring its key role in the forest carbon cycle, in agreement with previous studies (Pregitzer and Euskirchen, 2004; Fang et al., 2014; Du, 2015). Moreover, the forest NEP-age model developed for Xinjiang in this study is consistent with the model established by Gao et al (Gao et al., 2016). for the broader East Asia region, indicating a stable correlation between forest NEP and age. For future forest NEP studies, detrending the influence of forest age allows the contributions of climate factors to be more accurately compared across regions. This approach will enhance the ability of researchers to forecast the impacts of climate change on forest ecosystems and to characterize regional heterogeneity in forest carbon dynamics.

At regional and interannual scales, water and heat conditions are typically represented by MAT and MAP. The correlation between MAT and forest NEP has been widely confirmed (Magnani et al., 2007; Piao et al., 2009; Loudermilk et al., 2013), whereas the use of MAP in statistical models for NEP estimation remains relatively limited. Xinjiang is an arid region, and the growing environments of coniferous and broadleaf forests differ markedly, making classification-based analyses necessary. After removing the effect of forest age, NEP in coniferous forests shows a significant correlation with MAT, while NEP in broadleaf forests is significantly correlated with MAP. This pattern primarily reflects the spatial distribution of forest types: coniferous forests are mostly located in mountainous regions, whereas broadleaf forests are concentrated in plains. Forest ecosystems in Xinjiang’s mountains exhibit pronounced responses to temperature changes. Mountain forests employ efficient water-use strategies, such as deep root systems, adaptive leaf morphology, and stomatal regulation, combined with dual recharge from terrain precipitation and snowmelt, which buffer spatial and temporal variations in precipitation (Ci and Zhang, 2017; Yao et al., 2021). Temperature, as the dominant regulatory factor, influences NEP by directly affecting the photosynthesis-respiration balance of plants and regulating soil microbial activity. High water-use efficiency in mountain forests further enhances their sensitivity to temperature, making NEP in these areas more temperature-driven. In contrast, in plains regions, precipitation is the main source of water for forest ecosystems, and interannual fluctuations in MAP strongly affect soil moisture availability and plant water resources (Mitchell et al., 2015; Liu et al., 2024). Although groundwater levels and lateral river infiltration provide some localized recharge, the arid-semi-arid climate results in insufficient precipitation with highly uneven seasonal distribution. Consequently, plains forests depend more on MAP than mountain forests (Chen et al., 2015; Sun et al., 2019). Studies indicate that in arid regions, when precipitation falls below 20% of the long-term average, forest NEP decreases significantly, while groundwater fluctuations contribute less than 10% to buffering (Xue et al., 2021). In extremely dry years, the combined effects of high temperatures and precipitation deficits severely limit photosynthetic efficiency, with water stress becoming the dominant factor controlling NEP. Therefore, in Xinjiang’s plains, precipitation is the primary determinant of forest productivity.

After removing the effect of forest age, the statistical correlation between forest NEP and nitrogen deposition in Xinjiang was substantially weakened. This suggests that forest age may be a key covariate influencing the apparent effect of nitrogen deposition on NEP. Possible explanations are closely linked to human activities. For example, agricultural fertilization and intensive livestock farming in certain areas release ammonia nitrogen into the atmosphere, while industrial activities in regions undergoing active afforestation may contribute to higher nitrogen deposition in young forests. However, due to limited observational data on nitrogen deposition and the complexity of its impact mechanisms, future controlled experiments at the county or even forest stand scale are needed to clarify the true contribution of nitrogen deposition to forest carbon sinks (Fleischer et al., 2015).

### 4.2 Accuracy evaluation of the effect of climate factors on NEP

**Figure 8:**
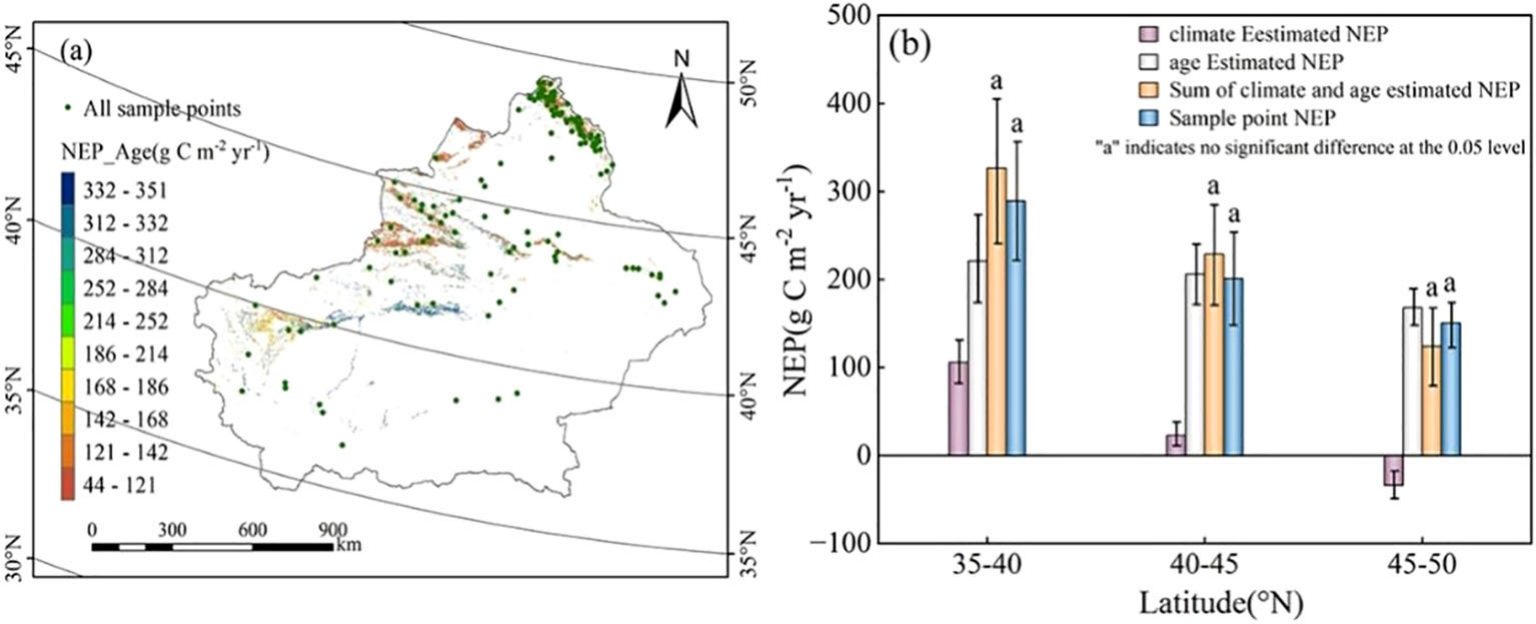
**(a)** Forests in Xinjiang divided by every 5° latitude, along with the forest NEP estimated by forest age and all forest sample plots (with 14, 47, and 68 sample plots in the three-dimensional regions, respectively); **(b)** The bar chart shows the estimated NEP values for Xinjiang forests in each 5° latitude region. The purple bars represent the NEP estimated by climate variables after removing the impact of forest age, the white bars represent the NEP estimated by forest age, the orange bars represent the total NEP estimated by forest age and climate factors, and the blue bars represent the observed NEP from the sample plots. The error bars indicate the standard error (with letter “a” indicating no significant difference at the 0.05 level).

We assessed the separate contributions of forest age and climate to NEP by decoupling their effects using Xinjiang forest plot data and associated datasets (latitude 33°-49°N). NEP contributions from forest age and from climate variables were estimated individually and then compared with the total NEP observed in the forest sample plots. Using Equations 1 and 2, forest age-driven NEP for Xinjiang was calculated (**Figure 8a**), while Equations 5 and 6 were used to estimate climate-driven NEP after removing the forest age trend (**Figure 6b**). Additionally, average NEP values for each 5° latitude band were calculated and presented in a bar chart (**Figure 8b**). Comparison with observed NEP from forest sample plots showed a high degree of agreement with the sum of the age- and climate-driven NEP, indicating that the method used in this study provides reliable estimates of NEP.

### 4.3 Uncertainty analysis of Xinjiang forest NEP estimation

The forest age and carbon flux data used in this study were derived from forest inventory sample plots and NEP product datasets. The reliability of forest NEP estimates is strongly influenced by the density and spatial distribution of sample plots. Older forests are primarily located in mountainous regions at higher elevations and are predominantly coniferous, whereas younger forests are mainly found in the Tarim River Basin and Ili River Valley, and are largely broadleaf. These regions also exhibit substantial differences in annual mean temperature and precipitation (Xiao et al., 2019).

**Figure 9:**
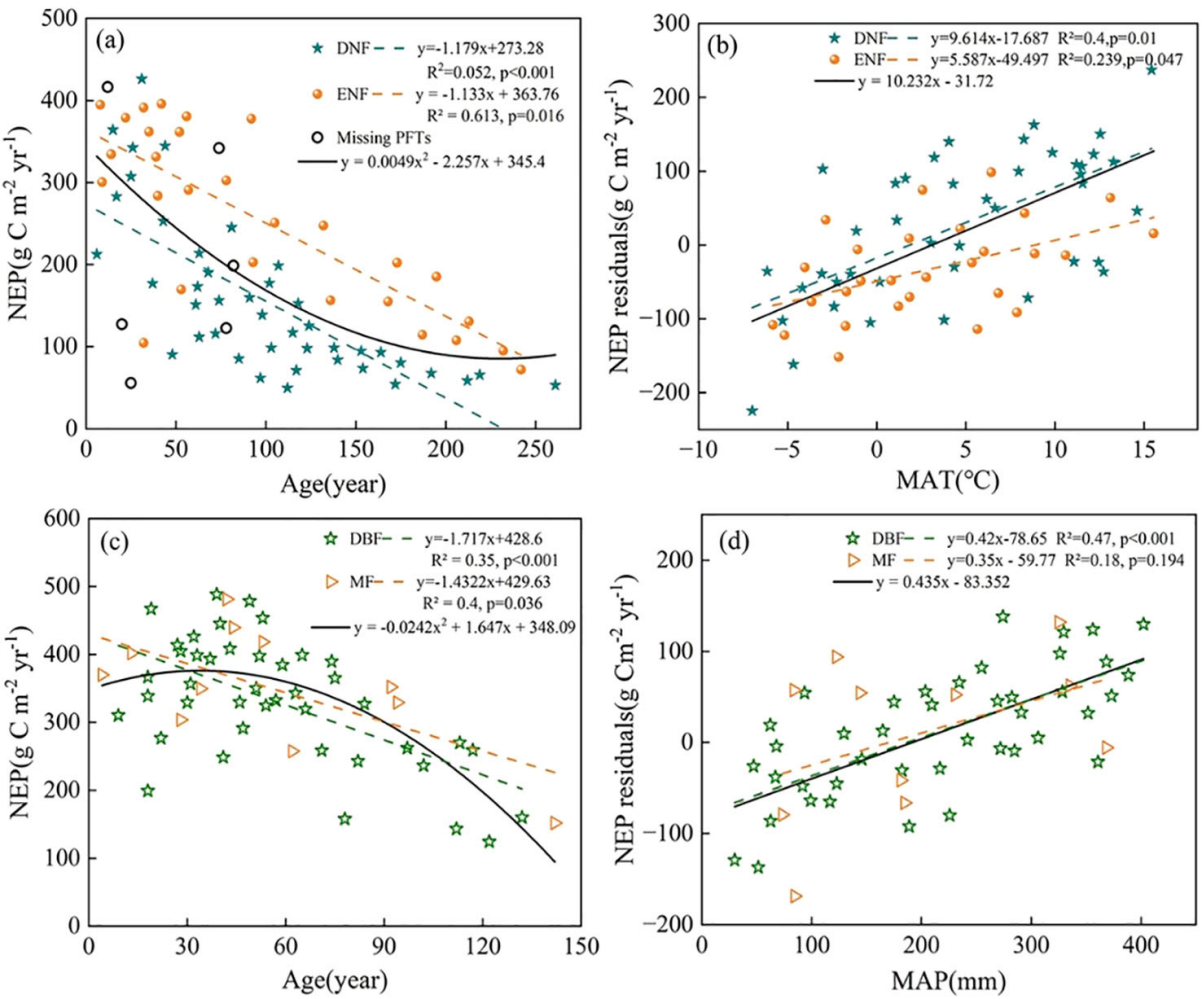
Differences in models between PFTs. **(a)** The difference in NEP with forest age between DNF and ENF in coniferous forests; **(b)** The difference in NEP residuals with MAT between DNF and ENF in coniferous forests; **(c)** The difference in NEP with forest age between DBF and MF in broadleaf and MFs; **(d)** The difference in NEP residuals with MAP between DBF and MF in broadleaf and MFs. DNF, ENF, DBF, and MF are abbreviations for deciduous conifer forest, evergreen conifer forest, deciduous broadleaf forest, and MF, respectively.

In addition, the aggregation of plant functional types (PFTs) can introduce uncertainty, as it may obscure or distort the true relationships between NEP, forest age, and climatic factors. To minimize this bias, data for each PFT should be analyzed independently prior to aggregation (Clark et al., 2011). In this study, a hierarchical classification scheme based on PFTs was adopted (Zhang et al., 2024). Coniferous forests were divided into DNF and ENF according to the key functional trait of leaf longevity (**Figures 9a, b**). Given the sparse distribution and small sample size of mixed conifer-broadleaf forests (n < 10% of total plots), these MFs were initially merged into the broadleaf category to avoid statistical bias. In subsequent analysis, MF were separated from broadleaf forests and treated independently from DBF (**Figures 9c, d**). This PFT-based functional heterogeneity analysis enhances the robustness of the relationships between NEP, forest age, and climate factors (**Figure 9**). Across PFTs, the general trend of NEP with forest age is consistent, with NEP declining as age increases, and differences in slopes are relatively small (**Figures 9a, c**). In coniferous forests, the average NEP of ENF is higher than that of DNF (**Figure 9a**); however, this difference may not solely result from PFT characteristics. DNF appears more sensitive to temperature increases during the growing season (**Figure 9b**), enabling efficient use of light and thermal resources, while its leaf shedding may enhance soil moisture retention. ENF, in contrast, responds more slowly to temperature changes due to its slower phenological adaptation, resulting in a delayed temperature response (Zhao et al., 2025). For broadleaf forests, the NEP residuals of DBF exhibit a strong correlation with MAP (R² = 0.47), a trend that largely mirrors the overall broadleaf forest pattern prior to separating MF. In contrast, MF shows weaker and more scattered responses (R² = 0.18, **Figure 9d**), likely reflecting complex internal and external influences, such as differing water requirements and utilization efficiencies among species (Ziran et al., 2024). Despite this variability, the relatively large number of forest plots in the study region, combined with the wide range of forest ages, temperatures, and precipitation values, ensures that the overall patterns of NEP variation with forest age, MAT, and MAP are reliably captured at the ecosystem level.

### 4.4 Contribution of climate factors to Xinjiang forest NEP

After accounting for forest age, MAT and MAP explained 40.10% and 39.90% of the residual variation in NEP for coniferous and broadleaf forests, respectively. Young forests were found to be more sensitive to climate change, as indicated by greater variation in NEP residuals at earlier forest age stages (**Figures 3b, e**). Since 2000, Xinjiang has increased its forest cover from 1.90% to 5.06% through initiatives such as natural forest protection and conversion of farmland to forest, substantially expanding forested areas (Wu et al., 2014; Liu et al., 2019; Yin et al., 2022). These afforestation efforts have also increased the proportion of young forests, reducing the region’s average forest age. Concurrently, Xinjiang has experienced a “warming and wetting” trend: rising temperatures have enhanced evapotranspiration, while the monsoon edge effect has contributed to increased regional precipitation. This shift toward a wetter climate, coupled with more frequent extreme high temperatures and short-duration heavy rainfall events, underscores the significant impact of climate change in the region (Yao et al., 2021; Yao et al., 2022). Consequently, despite global warming, Xinjiang’s forests, particularly younger stands, retain considerable potential for carbon sequestration.

If the influence of forest age is not accounted for when estimating forest NEP, the effects of climatic factors are substantially overestimated. Specifically, the NEP of Xinjiang’s forests estimated based solely on climate factors, without removing the forest age trend, is more than nine times higher than the NEP calculated after controlling for forest age (**Figure 6**). This estimate is also considerably higher than that obtained using Zan’s method (Zan, 2016), which reported an average annual NEP of 83.870 g m^-2^ yr^-1^ (totaling 69.890 Tg C from 2000 to 2020). Zan’s method estimates forest NEP over a long-term period by integrating forest inventory data, meteorological variables (temperature and precipitation), atmospheric factors such as CO_2_ concentrations, and the process-based InTEC model, capturing contributions from both natural and anthropogenic factors. Using this as a reference, our results indicate that, after removing the influence of forest age, the contribution of climate factors to forest NEP accounts for 24.52% of the total NEP (69.890 Tg C).

## 5 Conclusion

Based on Xinjiang forest survey data, climate data 2000-2020, and NEP data, this study employed a residual analysis approach to decouple and evaluate the relative contributions of forest age and climate to forest NEP in Xinjiang. It also compared the spatial distribution of climatic contributions to NEP before and after removing the effects of forest age. The main conclusions are as follows:

1. Forest age is the primary factor regulating the dynamics of forest NEP in Xinjiang, accounting for 45-49% of NEP variation and confirming the central role of age structure in regional carbon sink assessments.
2. When forest age is held constant, climatic factors emerge as the primary determinants of NEP. In Xinjiang, their influence exhibits strong vegetation type dependency. After removing the long-term effects of forest age, MAT explains 40.10% of the residual variation in NEP for coniferous forests, whereas NEP in broadleaf forests is primarily regulated by MAP (R² = 0.399). Overall, the relative contribution of climatic factors to the total annual NEP in Xinjiang forests is estimated at 24.52%. The analysis also indicates that the effect of nitrogen deposition on NEP is strongly age-dependent, as the correlation between nitrogen deposition and NEP substantially weakens once the influence of forest age is removed.
3. Methodologically, this study shows that neglecting the effects of forest age can result in an approximately tenfold overestimation of the contributions of climatic factors to NEP. The proposed “forest age-climate” decoupling framework provides a robust methodological approach for accurately quantifying the independent contributions of different driving factors.

## Data Availability

The original contributions presented in the study are included in the article/supplementary material. Further inquiries can be directed to the corresponding author.

## References

[B1] Adzrago D. Williams F. (2023). Mediation analysis of mental health characteristics linking social needs to life satisfaction among immigrants. SSM - Population Health 24, 101522. doi: 10.1016/j.ssmph.2023.101522, PMID: 37822807 PMC10563063

[B2] Besnard S. Carvalhais N. Arain M. A. Black A. De Bruin S. Buchmann N. . (2018). Quantifying the effect of forest age in annual net forest carbon balance. Environ. Res. Lett. 13, 124018. doi: 10.1088/1748-9326/aaeaeb

[B3] Bragazza L. Freeman C. Jones T. Rydin H. Limpens J. Fenner N. . (2006). Atmospheric nitrogen deposition promotes carbon loss from peat bogs. Proc. Natl. Acad. Sci. 103, 19386–19389. doi: 10.1073/pnas.0606629104, PMID: 17151199 PMC1748235

[B4] Carvalhais N. Forkel M. Khomik M. Bellarby J. Jung M. Migliavacca M. . (2014). Global covariation of carbon turnover times with climate in terrestrial ecosystems. Nature 514, 213–217. doi: 10.1038/nature13731, PMID: 25252980

[B5] Chen J. M. Ju W. Cihlar J. Price D. Liu J. Chen W. . (2003). Spatial distribution of carbon sources and sinks in Canada’s forests. Tellus B: Chem. Phys. Meteorology 55, 622–641. doi: 10.1034/j.1600-0889.2003.00036.x, PMID: 38018958

[B6] Chen Y. Li W. Xu C. Ye Z. Chen Y. (2015). Desert riparian vegetation and groundwater in the lower reaches of the Tarim River basin. Environ. Earth Sci. 73, 547–558. doi: 10.1007/s12665-013-3002-y, PMID: 41800275

[B7] Chen Z. Qian Z. Huang B. Feng G. Sun G. (2025). Increased drought impacts on vegetation productivity in drylands under climate change. Geophysical Res. Lett. 52(13), e2025GL115616. doi: 10.1029/2025GL115616, PMID: 40890438

[B8] Chen Z. Yu G. Wang Q. (2020). Effects of climate and forest age on the ecosystem carbon exchange of afforestation. J. Forestry Res. 31, 365–374. doi: 10.1007/s11676-019-00946-5, PMID: 41800275

[B9] Chen Z. Zan M. Yang X. Dong Y. (2023). Prediction of forest vegetation carbon storage in xinjiang. J. Ecol. Environ. 32, 226–234. doi: 10.16258/j.cnki.1674-5906.2023.02.002

[B10] Ci H. Zhang Q. (2017). Study on spatial and temporal characteristics of NDVI and its influence on climate change in Xinjiang. J. Earth Inf. Sci. 19, 662–671. doi: 10.3724/SP.J.1047.2017.00662, PMID: 41207781

[B11] Clark J. S. Bell D. M. Hersh M. H. Kwit M. C. Moran E. Salk C. . (2011). Individual-scale variation, species-scale differences: inference needed to understand diversity. Ecol. Lett. 14(12), 1273–1287. doi: 10.1111/j.1461-0248.2011.01685.x, PMID: 21978194

[B12] Coursolle C. Margolis H. A. Giasson M. A. Bernier P. Y. Amiro B. D. Arain M. A. . (2012). Influence of stand age on the magnitude and seasonality of carbon fluxes in Canadian forests. Agric. For. Meteorology 165, 136–148. doi: 10.1016/j.agrformet.2012.06.011, PMID: 41800429

[B13] Dragoni D. Schmid H. Wayson C. A. Potter H. Grimmond C. S. B. Randolph J. C. (2011). Evidence of increased net ecosystem productivity associated with a longer vegetated season in a deciduous forest in south-central Indiana, USA. Global Change Biol. 17, 886–897. doi: 10.1111/j.1365-2486.2010.02281.x, PMID: 41778641

[B14] Du E. (2015). Uncertain effects of nutrient availability on global forest carbon balance. Nat. Climate Change 5, 958–959. doi: 10.1038/nclimate2792, PMID: 41786825

[B15] Fang J. Guo Z. Piao S. Chen A. (2007). Terrestrial vegetation carbon sinks in China, 1981-2000. Sci. China Ser. D: Earth Sci. 50, 1341–1350. doi: 10.1007/s11430-007-0049-1, PMID: 41800275

[B16] Fang J. Kato T. Guo Z. Yang Y. Houghton R. A. (2014). Evidence for environmentally enhanced forest growth. Proc. Natl. Acad. Sci. U.S.A. 111, 9527–9532. doi: 10.1073/pnas.1402333111, PMID: 24979781 PMC4084460

[B17] Fleischer K. Wårlind D. Van Der Molen M. K. Rebel K. T. Arneth A. Erisman J. W. . (2015). Low historical nitrogen deposition effect on carbon sequestration in the boreal zone. J. Geophysical Research: Biogeosciences 120, 2542–2561. doi: 10.1002/2015JG002988, PMID: 41800289

[B18] Gao S. Zhou T. Zhao X. Wu D. Li Z. Wu H. . (2016). Age and climate contribution to observed forest carbon sinks in East Asia. Environ. Res. Lett. 11, 034021. doi: 10.1088/1748-9326/11/3/034021

[B19] Genet H. Bréda N. Dufrêne E. (2009). Age-related variation in carbon allocation at tree and stand scales in beech (Fagus sylvatica L.) and sessile oak (Quercus petraea (Matt.) Liebl.) using a chronosequence approach. Tree Physiol. 30, 177–192. doi: 10.1093/treephys/tpp105, PMID: 20018984

[B20] Hu L. Xiaofeng W. Shujiang C. Ping H. (2005). Dynamic analysis and evaluation of Xinjiang forest resources: Based on RS and GIS. J. Geographical Sci. 15, 346–352. doi: 10.1007/BF02837522, PMID: 41800275

[B21] Jia Y. Wang Q. Zhu J. Chen Z. He N. Yu G. (2021). Data set of spatial and temporal patterns of atmospheric inorganic nitrogen wet deposition in China from 1996 to 2015. China Sci. Data (Chinese English Online edition) 6, 9. doi: 10.11922/sciencedb.607, PMID: 35680932

[B22] Jianwu . (2014). Steeper declines in forest photosynthesis than respiration explain age-driven decreases in forest growth. Proc. Natl. Acad. Sci. United States America 111(24), 8856–8860. doi: 10.1073/pnas.1320761111, PMID: 24889643 PMC4066488

[B23] Leng Y. Li W. Ciais P. Sun M. Zhu L. Yue C. . (2024). Forest aging limits future carbon sink in China. One Earth 7, 822–834. doi: 10.1016/j.oneear.2024.04.011, PMID: 41800429

[B24] Li T. Liu G. (2014). Age-related changes of carbon accumulation and allocation in plants and soil of black locust forest on Loess Plateau in Ansai County, Shaanxi Province of China. Chin. Geographical Sci. 24, 414–422. doi: 10.1007/s11769-014-0704-3, PMID: 41800275

[B25] Liang Q. Chen Y. Duan W. Wang C. Li Y. Zhu J. (2025). Temporal and spatial changes of extreme precipitation and its related large-scale climate mechanisms in the arid region of Northwest China during 1961-2022. J. Hydrology 658. doi: 10.1016/j.jhydrol.2025.133182, PMID: 41800429

[B26] Lin Q. Zhu J. Wang Q. Zhang Q. Yu G. (2024). Patterns and drivers of atmospheric nitrogen deposition retention in global forests. Global Change Biol. 30, e17410. doi: 10.1111/gcb.17410, PMID: 38978457

[B27] Liu Y. Chen R. Han C. Liu Z. Zhao Y. Yang Z. (2024). Research on the characteristics, driving mechanism and spatial pattern of carbon sink in alpine ecosystem: A study case of Qilian Mountains. Agric. For. Meteorology 356, 110166. doi: 10.1016/j.agrformet.2024.110166, PMID: 41800429

[B28] Liu Y. Xiao Z. Zhang H. Yao B. Huang J. (2019). Analysis of forest land type change in Altai Mountains Natural Forest Protection Project area, Xinjiang from 2000 to 2016. For. resource Manage., 70–77. doi: 10.13466/j.cnki.lyzygl.2019.01.012

[B29] Loudermilk E. L. Scheller R. M. Weisberg P. J. Yang J. Dilts T. E. Karam S. L. . (2013). Carbon dynamics in the future forest: the importance of long-term successional legacy and climate-fire interactions. Global Change Biol. 19, 3502–3515. doi: 10.1111/gcb.12310, PMID: 23821586

[B30] Lu Y. Mu C. Gao X. Liang D. (2023). Effects of forest type and stand age on ecosystem carbon storage of plantations in Nenjiang Sandy Land of northeastern China. J. Beijing Forestry Univ. 45, 16–27. doi: 10.12171/j.1000-1522.20220294

[B31] Magnani F. Mencuccini M. Borghetti M. Berbigier P. Berninger F. Delzon S. . (2007). The human footprint in the carbon cycle of temperate and boreal forests. Nature 447, 849–851. doi: 10.1038/nature05847, PMID: 17568744

[B32] Mitchell S. R. Emanuel R. E. McGlynn B. L. (2015). Land-atmosphere carbon and water flux relationships to vapor pressure deficit, soil moisture, and stream flow. Agric. For. Meteorology 208, 108–117. doi: 10.1016/j.agrformet.2015.04.003, PMID: 41800429

[B33] Peng S. Ding Y. Liu W. Li Z. (2019). 1 km monthly temperature and precipitation dataset for China from 1901 to 2017. Earth Syst. Sci. Data 11, 1931–1946. doi: 10.5194/essd-11-1931-2019, PMID: 38859159

[B34] Piao S. Friedlingstein Ciais Peylin Zhu B. Reichstein M. (2009). Footprint of temperature changes in the temperate and boreal forest carbon balance. Geophysical Res. Lett. 36(7). doi: 10.1029/2009GL037381, PMID: 40890438

[B35] Pregitzer K. S. Euskirchen E. S. (2004). Carbon cycling and storage in world forests: biome patterns related to forest age. Global Change Biol. 10, 2052–2077. doi: 10.1111/j.1365-2486.2004.00866.x, PMID: 41778641

[B36] Qiu Z. Feng Z. Song Y. Li M. (2020). and Zhang, Carbon sequestration potential of forest vegetation in China from 2003 to 2050: Predicting forest vegetation growth based on climate and the environment. J. Cleaner Production 252, 119715. doi: 10.1016/j.jclepro.2019.119715, PMID: 41800429

[B37] Reay D. S. Dentener F. Grace S. J. Feely R. A. (2008). Global nitrogen deposition and carbon sinks. Nat. Geosci. 1, 430–437. doi: 10.1038/ngeo230, PMID: 41786825

[B38] Schulte-Uebbing L. F. Ros G. H. de Vries W. (2022). Experimental evidence shows minor contribution of nitrogen deposition to global forest carbon sequestration. Global Change Biol. 28, 899–917. doi: 10.1111/gcb.15960, PMID: 34699094 PMC9299138

[B39] Shan R. Feng G. Lin Y. Ma Z. (2025). Temporal stability of forest productivity declines over stand age at multiple spatial scales. Nat. Commun. 16(1), 2745. doi: 10.1038/s41467-025-57984-3, PMID: 40113748 PMC11926224

[B40] Su L. . (2024). Research progress on the effects of nitrogen addition on the storage and CO2 emission of forest soil organic carbon pool. Acta Ecologica Sin. 44, 2717–2733. doi: 10.20103/j.stxb.202305250867

[B41] Sun W. Wang Y. Fu Y. H. Xue B. Wang G. Yu J. . (2019). Spatial heterogeneity of changes in vegetation growth and their driving forces based on satellite observations of the Yarlung Zangbo River Basin in the Tibetan Plateau. J. Hydrology 574, 324–332. doi: 10.1016/j.jhydrol.2019.04.043, PMID: 41800429

[B42] Sutton M. A. Simpson D. Levy P. E. Smith R. I. Reis S. Van Oijen M. . (2008). Uncertainties in the relationship between atmospheric nitrogen deposition and forest carbon sequestration. Global Change Biol. 14, 2057–2063. doi: 10.1111/j.1365-2486.2008.01636.x, PMID: 41778641

[B43] Yuan W. Liu S. Yu G. Bonnefond J. M. Chen J. Davis K. . (2010). Global estimates of evapotranspiration and gross primary production based on MODIS and global meteorology data. Remote Sens. Environ. 114, 1416–1431. doi: 10.1016/j.rse.2010.01.022, PMID: 41800429

[B44] Wu B. . (2014). Land cover change in China during the first decade of the 21st century. quaternary Res. 34, 723–731. doi: JournalArticle/5b434737c095d716a4c30062

[B45] Xiao Z. Zhang Y. Li J. (2019). Value assessment of forest ecosystem services in Xinjiang. J. Xinjiang Univ. (Natural Sci. Edition) 36, 483–490. doi: 10.13568/j.cnki.651094.2019.04.017

[B46] Xu L. Saatchi S. S. Shapiro A. Meyer V. Ferraz A. Yang Y. . (2017). Spatial distribution of carbon stored in forests of the Democratic Republic of Congo. Sci. Rep. 7, 15030. doi: 10.1038/s41598-017-15050-z, PMID: 29118358 PMC5678085

[B47] Xue L. Fu F. Chen X. Liu Y. Han Q. Liao S. . (2021). Analysis on water use efficiency of Populus euphratica forest ecosystem in arid area. Theor. Appl. Climatology 145, 717–730. doi: 10.1007/s00704-021-03636-7, PMID: 41800275

[B48] Yao J. Chen J. Nur D. Han X. Mao W. (2021). Consideration on climate and hydrological change trend and problems in Xinjiang. Permafrost 43, 1498–1511. doi: 10.7522/j.issn.1000-0240.2021.0101

[B49] Yao J. Li M. Nur D. Chen J. Mao W. (2022). The characteristics of warm and humid climate in Xinjiang at different time scales. Arid zone Stud. 39, 333–346. doi: 10.13866/j.azr.2022.02.01

[B50] Yi C. Ricciuto D. Li R. Wolbeck J. Xu X. Nilsson M. . (2010). Climate control of terrestrial carbon exchange across biomes and continents. Environ. Res. Lett. 5, 034007. doi: 10.1088/1748-9326/5/3/034007

[B51] Yin Z. Feng Q. Wang L. Chen Z. Chang Y. Zhu R. (2022). Vegetation cover change and its influencing factors in northwest China from 2000 to 2019. Desert China 42, 11–21, no. doi: 10.7522/j.issn.1000-694X.2021.00200

[B52] Yu G. Chen Z. Piao S. Peng C. Ciais P. Wang Q. . (2014). High carbon dioxide uptake by subtropical forest ecosystems in the East Asian monsoon region. Proc. Natl. Acad. Sci. United States America 111, 4910–4915. doi: 10.1073/pnas.1317065111, PMID: 24639529 PMC3977309

[B53] Yu Z. Liu S. Li H. Liang J. Liu W. Piao S. . (2024). Maximizing carbon sequestration potential in Chinese forests through optimal management. Nat. Commun. 15, 3154. doi: 10.1038/s41467-024-47143-5, PMID: 38605043 PMC11009231

[B54] Zan M. (2016). Temporal and spatial variation characteristics of forest carbon flux and carbon storage in Xinjiang and its main influencing factors. 2016. doi: 10.27235/d.cnki.gnjiu.2016.001448

[B55] Zhai L. . (2025). Time-series forest age estimation in Xinjiang based on forest disturbance and recovery detection. Ecol. Indic. 170, 113043. doi: 10.1016/j.ecolind.2024.113043, PMID: 41800429

[B56] Zhang Z. . (2024). Estimation of terrestrial carbon sink in China combining bottom-up and top-down methods. Climate Environ. Res. 29, 229–242. doi: 10.3878/j.issn.1006-9585.2024.23152

[B57] Zhao J. Yu Y. Hu Y. Beyer M. Zhang J. (2025). Measurement and modeling of canopy interception loss of evergreen, deciduous and mixed forests in a subhumid watershed on the Loess Plateau, China. J. Hydrology 654, 132820. doi: 10.1016/j.jhydrol.2025.132820, PMID: 41800429

[B58] Zhou T. Shi P. Jia G. Dai Y. Zhao X. Shangguan W. . (2015). Age-dependent forest carbon sink: Estimation via inverse modeling. J. Geophysical Res. Biogeosciences 120(12), 2473–2492. doi: 10.1002/2015JG002943, PMID: 41800289

[B59] Zhou K. Xu W. Zhang L. Ma M. Liu X. Zhao Y. (2023). Estimating nitrogen and sulfur deposition across China during 2005 to 2020 based on multiple statistical models. Atmos. Chem. Phys. 23, 8531–8551. doi: 10.5194/acp-23-8531-2023, PMID: 38859159

[B60] Zhu X.-J. . (2014). Geographical statistical assessments of carbon fluxes in terrestrial ecosystems of China: Results from upscaling network observations. Global Planetary Change 118, 52–61. doi: 10.1016/j.gloplacha.2014.04.003, PMID: 41800429

[B61] Zhu X. J. Yu G. R. He H. L. Wang Q. F. Chen Z. Gao Y. N. (2021). Effect of atmospheric nitrogen deposition and its components on carbon flux in terrestrial ecosystems in China. Environ. Res. Lett. 202, 111787. doi: 10.1016/j.envres.2021.111787, PMID: 34339690

[B62] Ziran L. Xin W. Xinxiao Y. Guodong J. (2024). Seasonal variation of water utilization sources and responses to precipitation in the dominant species of broad-leaved and needle-leaved mixed forests in Mount Lushan, China. Chinese Journal of Applied Ecology/Yingyong Shengtai Xuebao 35(4). doi: 10.13287/j.1001-9332.202404.015, PMID: 38884223

[B63] Zuur A. F. Ieno E. N. Elphick C. S. (2010). A protocol for data exploration to avoid common statistical problems. Methods Ecol. Evol. 1, 3–14. doi: 10.1111/j.2041-210X.2009.00001.x, PMID: 41778641

